# The transformation of masculinity orientations and work-related attitudes in men treated for depression (TRANSMODE): study protocol for a mixed-methods observational study

**DOI:** 10.1186/s12888-023-04979-3

**Published:** 2023-07-10

**Authors:** Silvia Krumm, Gironimo Krieg, Natalie Lamp, Franziska Marek, Paul Nickel, Maria Panzirsch, Maja Stiawa, Petra Beschoner, Peter Brieger, Karel Frasch, Marcus Gertzen, Harald Gündel, Alkomiet Hasan, Markus Jäger, Paulo Kling-Lourenco, José Marie Koussemou, Raimund Steber, Reinhold Kilian

**Affiliations:** 1 Department of Psychiatry and Psychotherapy II, Ulm University, Ulm, Germany; 2 Department of Psychiatry, Psychotherapy and Psychosomatics, Bezirkskrankenhaus Donauwörth, Donauwörth, Germany; 3 Department of Psychosomatic Medicine and Psychotherapy, Christophsbad Clinic, Göppingen, Germany; 4 Psychiatry and Psychotherapy, Faculty of Medicine, University of Augsburg, Augsburg, Germany; 5 Department of Psychosomatic Medicine and Psychotherapy, Ulm University, Ulm, Germany; 6 Kbo-Isar-Amper Hospital, Region Munich, Germany; 7 Bezirkskrankenhaus Kempten, Kempten, Germany; 8 Clinic for Psychiatry, Psychotherapy and Psychosomatics, Heidenheim Clinic, Heidenheim, Germany; 9 Bezirkskrankenhaus Memmingen, Memmingen, Germany

**Keywords:** Depression, Men´s depression, Masculine orientations, Work-related attitudes, Mix-methods study, Latent transition analysis, Qualitative study, Partners of depressed men, Dyadic coping

## Abstract

**Background:**

Masculinity norms play a crucial role in men’s help-seeking behaviors, service-use, and coping strategies for depression. While previous studies provided evidence for the association between gender role orientations, work related attitudes, stigmatization of men with depression and depressive symptoms, it remains unclear to what extent gender role orientations change over time and whether psychiatric and psychotherapeutic treatment have an impact on these transformations. Additionally, the role of partners in supporting depressed men and the impact of dyadic coping on these processes have not been explored. The aim of this study is to investigate how masculinity orientations and work-related attitudes change over time in men treated for depression, and to examine the role of their partners and dyadic coping in these transformation processes.

**Methods:**

TRANSMODE is a prospective longitudinal mixed-methods study investigating the transformation of masculinity orientations and work-related attitudes in men treated for depression between the ages of 18 and 65 from different settings in Germany. The study will recruit 350 men from various settings for quantitative analysis. By applying a latent transition analysis, the primary outcome are changes in masculine orientations and work-related attitudes over time, measured at four times (t0, t1, t2, t3) with intervals of 6 months. Qualitative interview with a subsample of depressed men selected using latent profile analysis, will be conducted between t0 and t1 (a1) with a follow-up of 12 months (a2). In addition, qualitative interviews with the partners of depressed men will be conducted between t2 and t3 (p1). Qualitative data will be analysed using qualitative structured content analysis.

**Discussion:**

A comprehensive understanding of the transformation processes of masculinity orientations over time including the impact of psychiatric/psychotherapeutic treatment and the role of partners can lead to the development of gender-sensitive depression treatment tailored to the unique needs of men with depression. Thus, the study can promote more effective and successful treatment outcomes and further contribute to reducing the stigma surrounding mental health issues among men and encourage them for mental health service use.

**Trial registration:**

This study is registered in the German Clinical Trail Register (DRKS) and the WHO International Clinical Trials Registry Platform (ICTRP) under registration number DRKS00031065 (Date of registration 06 February 2023).

## Background

Masculinity norms have been identified as playing a crucial role in men’s coping with depressive symptoms and their willingness to seek professional help [1–5]. In the Gotland study, conducted between 1981 and 1990, Rutz et al. [6, 7] found that traditional masculinity norms explained the paradox that men had a lower frequency of depression [8] but a higher rate of suicides than women [7, 9]. Based on this, several authors developed the concept of male depression, suggesting that in men, depression occurs with different symptoms than those applied in psychiatric diagnosis classification systems such as ICD or DSM [6, 10–12]. Unlike depression in women, male depression is characterized by increased irritability, aggressiveness, anger and antisocial behavior [6, 10, 12, 13]. In addition to these differences, men were also found to mask classical symptoms of depression such as fatigue, sadness, and weariness to avoid being considered unmanly by their social environment [3].

Working conditions have been identified as an important factor in the development and course of depression among both men and women [14–21]. However, research suggests that the impact of work related problems on symptoms of depression may differ between men and women [21]. A study conducted in Canada by Wang et al. [21] showed that job insecurity was positively associated with depression in men but not in women. Similarly, Pyöriä et al. [22] found that precarious employment was associated with an increased risk of future depression in men but not in women in a sample of Finish salary earners. The authors of both studies suggested that men’s “masculine” self-identity might be more strongly linked to paid work and the role as breadwinner, while women’s self-identity might be more rooted in their social skills and their role as members of social networks [21, 22]. This interpretation was supported by results from the WHO mental health surveys, which revealed that the gender difference in depression decreased with decreasing levels of traditional masculinity orientations [8]. However, this interpretation was not supported by the results of a recent meta analyses, which suggested that the gender differences in major depression across countries increase with gender equity [23]. With regard to the male suicide rate, the gender role equity interpretation would be supported by a cross-country analysis of Reeves & Struckler [24], indicating that an increase in gender norm equity was associated with a decrease in the association between unemployment and the suicide rate in men but not in women.

While there is strong evidence that the adherence to traditional male gender norms serves as a barrier to seeking informal and formal help or support for mental health problems, limited knowledge exists about the impact of masculinity norms on post-diagnostic processes and pathways, including depression treatment and/or psychotherapy use. Findings on high drop-out rates among men suggest that men’s needs may not be adequately addressed within therapeutic settings. There is some evidence that men have ambivalent or even critical attitudes towards psychotherapy [25] or antidepressants [26]. An Australian survey revealed that men dropped out due to a lack of therapist-client connection and dissatisfaction with the therapy processes, with some men reporting feelings of emasculation in attending therapy [27]. Eggenberger and colleagues found that reduced psychotherapy use in men with experienced psychological distress was associated with higher self-identified masculinity [28]. However, endorsement of masculinity norms exerts a different impact on men’s mental health treatment utilization: while greater endorsement of the status norm is associated with increased utilization, endorsement of the anti-femininity norm and the toughness norm is associated with decreased service utilization [29]. From male service users’ perspectives, perceptions of autonomy and control in therapeutic interactions are crucial factors for treatment engagement or disengagement [30].

The growing evidence for the detrimental effects of men’s endorsement to traditional masculinity norms is mirrored in mental health professionals’ reflections on the impact of masculinity norms on mental health care. In a German qualitative study, mental health professionals described depressed male patients’ treatment expectations as different from females’ and characterized by preferences for biological explanatory models and “mechanical” approaches. Accordingly, mental health professionals reported addressing the negative effects of normative expectations regarding male gender on men’s mental health during depression treatment [31]. Based on experiences with men’s reluctance to engage in usual therapeutic environments favoring emotional rather than “problem-solving” coping approaches, Westwood & Black describe a specific approach to successfully engaging men in the counselling process by using appropriate language or pro-active approaches [32]. However, despite the efforts to adjust concepts of depression treatment to the particular needs of men, the impact of psychiatric and psychotherapeutic treatment on changing masculinity orientations or work role orientations of men with depression has not been investigated so far.

Beyond the potential impact of professional treatment, traditional gender roles can be changed or modified during the process of couples’ coping with illness. Couples in which a male partner is depressed have been found to co-construct alternative masculinities by recalibrating gender relations [33]. Due to their partners’ failure to correspond to traditional male roles, female partners make adaptations and take on responsibilities in order to “protect their partners and the façade of gender normalcy” [34]. Although earlier research has focused on women’s role as caregivers for diseased male partners [35, 36] there is only limited research on the involvement of partners in coping with men’s depression and the impact of dyadic coping on men’s masculinity orientations and work role orientations.

Several of the studies mentioned above suggest that a masculine self-identity in combination with a traditional job role orientation, may increase men’s vulnerability against adverse job or economic conditions. However, this hypothesis has not been investigated any further so far. Only recently, Kilian et al. [1], in a mixed-method study, identified traditional masculinity norms in combination with a traditional job role orientation as a cognitive pattern associated with an increased level of psychopathological symptoms and also with an increased level of stigmatizing and self-stigmatizing attitudes regarding depressive disorders in a sample of men treated for depression. In the qualitative part of the study, the authors found indications that some of the respondents started to identify their masculinity and work role orientations as a cause of depression and made efforts to change these dysfunctional attitudes [37, 38]. Due to the cross-sectional character of the study, the authors were not able to make conclusions about the causal direction of the associations between masculinity orientations and depressive symptoms and about the role of psychiatric and psychotherapeutic treatment in this association.

In order to better tailor existing depression treatment services for male patients, we need to better understand men’s experiences, attitudes and needs regarding depression therapy. Therefore, the authors designed a longitudinal mixed method study to observe men with depression over a period of 24 months with the purpose of understanding the interrelation between treatment and masculinity orientations.

## Methods/Design

### Study aim

The aim of the TRANSMODE study is to investigate the transformation of masculinity orientations and work-related attitudes in a sample of men treated for depression and their partners. The main objectives are:

1. To assess different latent classes of masculinity orientations and work-related attitudes of men treated for depression and the changes of the latent classes over time (transformation).
2. To investigate the interplay between the transformation of masculinity orientations and work-related attitudes and depressive symptoms, stigmatizing attitudes, and the use of psychiatric or psychotherapeutic treatment.
3. To investigate the subjective perspectives of men treated for depression on the transformation process and the subjective perceptions of the impact of the treatment on the masculinity orientations and work-related attitudes and vice versa.
4. To explore the subjective perspectives of depressed men’s parters on their partner’s depression and on the co-construction of masculinity orientations and work-related attitudes.

### Study design

This protocol describes a prospective mixed-methods observational study consisting of three parts (subprojects). The mixed-methods-design is realized through the combination of quantitative surveys and qualitative guided interviews and based on the Explanatory Sequential Design [38, 39]. In addition, due to the longitudinal design, quantitative and qualitative data are collected in parallel [40].

- As the first part, a quantitative longitudinal observational study including 350 men treated for depression will be conducted with four measurement points over 24 months (subproject 1).
- As the second part, a qualitative longitudinal study will be conducted including a subsample of approximately 60 participants with two assessement points over 12 months (subproject 2).
- As the third part, a cross-sectional qualitative study will be conducted, involving appr. 15 partners of the male participants who have been selected for the qualitative longitudinal study (subproject 3).

### Study setting

Study participants will be recruited at the following study sites providing in- and outpatient mental health treatment and care in Germany:

- Department of Psychiatry and Psychotherapy II, Ulm University, Bezirkskrankenhaus Guenzburg.
- Psychosomatic Medicine and Psychotherapy, University Hospital Ulm.
- Department of Psychiatry, Psychotherapy and Psychosomatics at the University of Augsburg, Bezirkskrankenhaus Augsburg.
- District Hospital Kempten, Bezirkskrankenhaus Kempten.
- District Hospital Memmingen, Bezirkskrankenhaus Memmingen.
- District Hospital Donauwörth, Bezirkskrankenhaus Donauwörth.
- Kbo-Isar-Amper Hospital, München, Haar, Fürstenfeldbruck.
- Clinic for Psychiatry, Psychotherapy and Psychosomatics, Heidenheim Clinic.
- Clinic of Psychiatry, Psychotherapy and Psychosomatic, Imland Klinik Rendsburg.

Furthermore, participants will be recruited via social media (Facebook and Instagram) using paid advertisements, with each advertisement shown to approximately 10.000 men. In addition, recruitment will take place within self-help groups for people with depression, particularly in the south of Germany, but also nationwide.

### Participants and eligibility criteria

Men between the ages 18 and 65 who have been diagnosed with depression and have received treatment for depression in the last 12 months, or are about to receive their first treatment, will be included in the study. Men with a primary diagnosis of an addiction disorder will be excluded. Eligibility criteria will be checked by study workers prior to study inclusion using a screening tool. For inpatients and day hospital patients, the screening will be conducted by the treating physician or psychotherapist.

### Participant timeline

**Fig. 1:**
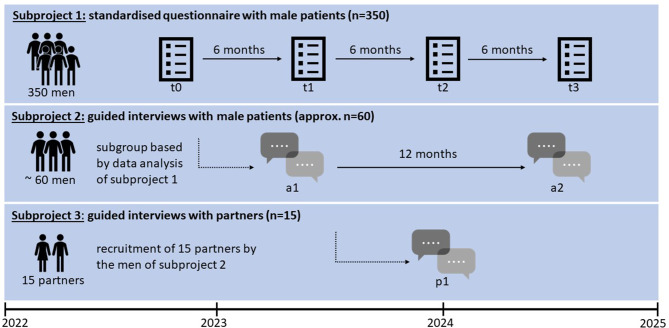
Participant timeline

The recruitment period started in May 2022 and continues until June 2023, and the participation period for each participant will be 24 months. Surveys for the quantitative subproject will be conducted at T0 (baseline) and with intervals of 6 months (t0; t1 = t0 + 6months, t2 = t1 + 6months, t3 = t2 + 6months). The qualitative interviews with the participants will be conducted between t0 and t1 (a1) with a follow-up after 12 months (a2). The qualitative interviews with the partners will be conducted between t2 and t3 (p1). The quantitative measurement, resp. qualitative assessement time points of the 3 subprojects are shown in Fig. 1. At the time of the submission of this protocol, n = 324 have been included in the study.

### Outcomes

#### Subproject 1 (quantitative longitudinal observational study)

##### Primary outcome

Primary outcome of subproject 1 is the transition of participants between latent class membership related to masculinity orientations (using MRNS) and work-related attitudes (using the AVEM) between the follow-ups. Data will be collected at t0, t1, t2, t3 at intervals of 6 months.

##### Secondary outcomes

The secondary oucomes include the change in stigmatizing attitudes towards mental illness (using the DSS), the change in depressive symtoms (using the PHQ SADS), characteristics of the treatment process (using the CSSRI) and sociodemographic characteristics in regard with transition between latent classes of masculinity orientations (using the MRNS) and work-related attitudes (using the AVEM), measured at t0, t1, t2, and t3.

#### Subproject 2 (qualitative longitudinal study)

The qualitative study investigates the subjective perceptions of the transformation processes described above at two assessement time points (a1 and a2). Particular attention will be drawn to factors that might affect the changes and the role attributed to depression treatment, such as in the context of psychotherapy and psychiatric or other medical treatment. Furthermore, we will explore (a) how knowledge about depression and therapy is integrated into experiential and everyday knowledge, (b) how this integration enables a transformation towards health-promoting masculinity orientations and positive attitudes towards occupational health and (c) how this affects illness and recovery processes including self-interpretation, world-interpretation, and daily life. In addition, we aim at identifying treatment elements that are perceived as conducive or obstructive for recovery, and the ways in which male gender norms can impact individuals experiencing depression and its treatment.

#### Subproject 3 (cross-sectional qualitative study)

The qualitative study explores the subjective perspectives of the partners of men treated for depression through problem-centered interviews between t2 and t3 (p1) with (female and male) partners of the participants of subprojects 1 and 2. Particular attention will be paid to the partners’ views on changes in their partner’s masculinity orientations and work-related attitudes occurred during the therapy or recovery processes, and on the negotiations of gender roles within the relationship (co-construction of masculinity, or co-coping) within partnerships.

### Recruitment and sample size calculation

#### Subproject 1 (quantitative longitudinal observational study)

A necessary sample size of n = 250 for the quantitative study has be calculated on the basis of simulation studies with regard to the reliability of latent class assignment and latent transition probabilities with a minimal power of 0.80 [39]. Taking into account a total dropout rate of 30%, a sample size of 350 participants has been targeted for inclusion. The recruitment will be conducted from May 2022 to June 2023 in clinical settings, via patient organisations and via social media. For each inpatient setting, clinical staff members have been appointed as contact persons by the chief doctors. Clinical contact persons have been informed about the study details and the eligibility criteria. In addition, information flyers have been laid out at the common hospital areas. Clinical staff members contacted potentially eligible patients and asked them for their consent to pass contact information to research workers. Patient organisations have been contacted, informed about the study, and provided with study flyers for distribution to members by e-mail. Self-help groups that specialise in men’ groups have also been contacted. Participants have been called on to participate at regular intervals via various media, including payed advertisements in social media (Facebook, Instagram), local newspapers, radio and online pages of the BKH clinics and University Ulm.

#### Subproject 2 (qualitative longitudinal study)

Subproject 2 includes two assessement time points (a1, a2) and uses a mixed-method approach to select the subsample [40]. Using the results of the interims latent class analysis after t0, n = 60 men will be recruited from the subproject 1 sample according to the explored classes. Based on our experience from the MenDe study [1], we assume a 3-class model. At least n = 20 men from each class (class representatives) will be interviewed, resulting in a total number of n = 60 qualitative interviews conducted between t0 and t1 (a1). This high number is necessary in order to ensure a sufficiently large number of subjects for the follow-up interview after 12 months (a2) in case of a maximum drop-out rate of 50%. The representatives of each class will preferably be selected according to being in the early stages of depression treatment and equally distributed in regard with age. The repeat survey is conducted 12 months after baseline (a1) with the aim of n = 30 participants agreeing for the follow-up interview. In summary, over the two assessment time points, the aim is to conduct 90 interviews in total (a1: n = 60, a2: n = 30). In case the drop-out number is higher than expected and/or additional interviews are necessary to reach theoretical saturation [41], the recruitment of participants for a2 will be expanded to the full sample of subproject 1.

#### Subproject 3 (cross-sectional qualitative study)

Participants for subproject 3 are recruited at a1 if participants of subproject 2 indicate that they are living in a partnership. Depending on their consent to contact their partners for study participation, the partners will be invited to participate in the qualitative interview at p1. Assuming a participation rate of 50% of all partners, a sample size of n = 15 is targeted. If the recruitment does not yield enough partners, the recruitment will be extended to the full sample of subproject 1. The final sample size will be determined by theoretical saturation and may be adapted based on the results of the interviews [41].

### Data collection and study instruments

#### Subproject 1 (quantitative longitudinal observational study)

The quantitative data will be collected using the SoSci Survey online platform (SoSci Survey GmbH, Munich). Participants receive the access data for the questionnaire. If online participation is not wished or possible, the participants receive the paper version of the questionnaires together with stamped and addressed return envelopes. The following assessments will be used:

- Male Role Norm Scale (MRNS) is a widely used and validated tool for the assessment of masculine gender role attitudes and consists of 22 items assessing attitudes towards traditional masculine norms including self-reliance, toughness, and dominance. Respondents can rate their agreement on a Likert scale ranging from “strongly disagree” to “strongly agree”.
- Work-Related Behaviour and Experience Patterns (AVEM) measures work-related behaviour and experience pattern with eleven dimensions including professional ambition, offensive problem solving or experience of success at work. Respondents can rate their agreement on a five level Likert scale ranging from “strongly disagree” to “strongly agree” [42].
- Depression Stigma Scale (DSS) is used to measure stigma associated with depression. It includes two subscales measuring two different types of stigma: personal and perceived stigma. Respondents can rate to each item on a five-point scale ranging from “strongly disagree” to “strongly agree”. Higher scores indicate higher levels of depression stigma [43].
- The Patient Health Questionnaire will be used to assess somatic and psychosocial stress [44]. The first question covers 12 somatic complaints in the last 4 weeks and respondents can rate on a scale ranging from “not affected”, “slightly affected”, and “severely affected”. The following 16 points cover mental impairments and participants can choose between: “not at all”, “on individual days”, “on more than half of the days”, and “almost every day”. The last item assess difficulties to cope with work or household chores based on the symptoms mentioned and respondents can rate on a four-point scale ranging from “not difficult at all” to “very difficult”.
- The Bem Sex Role Inventory (BSRI) will be used to assess gender role self-concept in relation to masculine and feminine personality traits. It consists of 60 items and respondents can rate each item on a five-point scale ranging from “strongly disagree” to “strongly agree” [45].
- The Client Sociodemographic and Service Receipt Interview (CSSRI) assesses social and demographic data, the use of medication, utilisation of health care services and allows to estimate healthcare costs using a total of 83 items [46].

#### Subproject 2 (qualitative longitudinal study)

Selected participants of subproject 1 who consented to be interviewed in the qualitative study part will be contacted and asked for participation. Problem-centred interviews will be conducted via video telephony by using an encrypted provider which complies with the requirements of the General Data Protection Regulation in Germany (DSGVO) and requirements for medical data. If an online participation is not possible or not wished by the participant, a face-to-face interview is offered. The interviews will be conducted by researchers trained and experienced in qualitative interviewing. For the first interview (a1), a semi-structured interview guide has been developed including the following topics: Treatment start; context of depression; course of treatment; social environment and disclosure; work, gender, society, and associations with depression. A pilot interview will be conducted to evaluate the interview guide, and to modify if necessary. The interview guide is developed by the research group, based on relevant literature and experiences gained from the previous MenDe study [1, 2, 31, 47]. For further validation, the interview guide is discussed in qualitative research workshops including researchers from diverse mental health research fields. For the second interview (a2), an interview guide is developed, focusing on potential changes in regard with treatment and recovery processes. The topics include subjective perception of the therapy, changes in illness theory, changes in work and family situation, changes in disclosure and changes in self-image.

#### Subproject 3 (cross-sectional qualitative study)

The qualitative data will be collected using problem-centred interviews. Based on the (preliminary) findings from subproject 1 and 2 as well as relevant literature, we will develop an interview guide in order to explore partners’ subjective perception of changes in regard to therapy and recovery processes of their male partners, gender and work-related attitudes, masculinity orientations and its impact on the relationship. The interview guide covers various topics such as communication within the partnership, dealing with the partner’s depression, and the effects of depression on the partner and the partnership. The interview guide is developed similar to subproject 2. We will conduct a pilot interview in order to evaluate the interview guide and to modify if necessary.

### Promotion of study participation

In subproject 1, participants are contacted and informed two weeks before the next quantitative survey takes place. Participants receive the access data for the questionnaire by e-mail. The participants are asked to enter their data within four weeks, starting two weeks before the measurement time point. For each survey, the participants receive remuneration of 10 €.

In subproject 2, we will contact participants and inform them two weeks prior to the follow-up interview to make an appointment. Participants receive a link for a video-call by e-mail. For each interview, participants receive remuneration of 30 €.

In subproject 3, participants will be contacted after inclusion of participants of subproject 2 for the qualitative repeat survey (a2). Participants receive a link for a video-call by e-mail. For each interview, a remuneration of 30 € is provided.

### Data management

Data entry into the SociSurvey data base is possible with any device with internet access. Data will be checked for completeness. The collected data will be visible to the study team in pseudonymised and encoded forms. The data manager will back up the data at regular intervals as a csv file. Plausibility checks and generation of new variables like sum scores will be carried out with the statistical software SPSS version 28. The video interviews will be audio recorded and transcribed for analysis. For the transcription, f4x will be used, an online service to transcript audio recordings which complies with the General Data Protection Regulation in Germany (DSGVO) and requirements for medical data. Subsequently, the transcripts will be revised and pseudonymised by a research worker.

### Data analysis

#### Statistical methods (subproject 1)

**Fig. 2:**
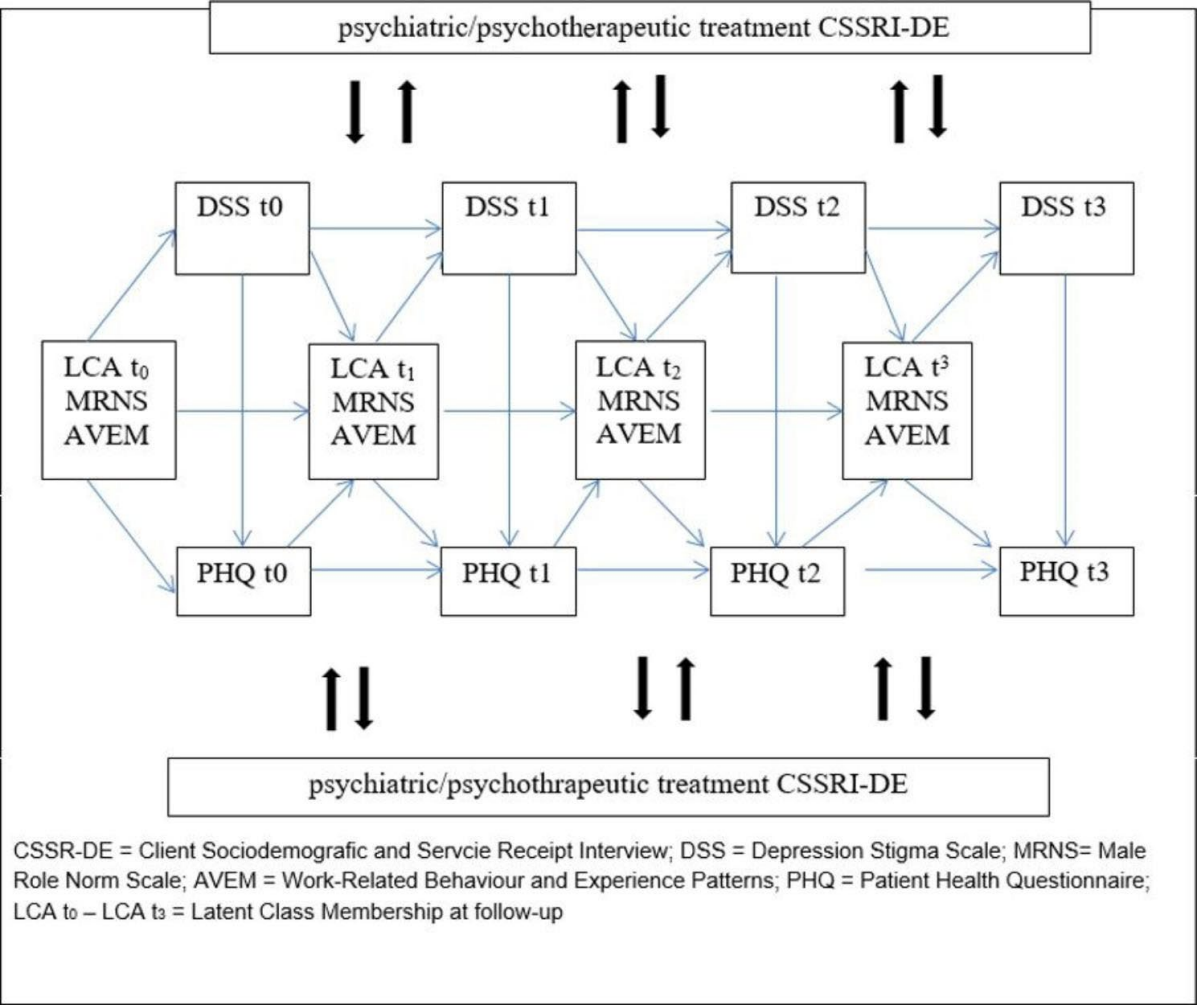
Cross-correlational longitudinal path model of latent class membership

A latent transition analysis (LTA) is chosen as the statistical analysis model [48]. The LTA is an extension of the Latent Class Analysis (LCA) for longitudinal data. Using the LTA, transition probabilities between latent class assignments can be determined over several measurement times and the effects of covariates on these transition probabilities can be estimated. In order to analyse the importance of treatment pathways and psychiatric / psychotherapeutic treatment elements, the data from the treatment anamnesis and the CSSRI surveys are categorized and integrated into the analysis model. All regression models are controlled for sociodemographic characteristics and disease duration (Fig. 2).

#### Qualitative data analysis (subproject 2, subproject 3)

The interviews will be analysed using both qualitative structured content analysis according to Kuckartz [49] and reconstructive analysis according to Kruse [50]. For data management MAXQDA analysis software will be used. In the first step of the content analysis, the transcripts of the first interviews (10–25%) will be coded deductively based on the interview guide topics. Iteratively, the deductive codes and categories will be extended, refined or modified by means of inductive coding. In the second step, the preliminary coding tree will be applied to the entire material and, if necessary, further extended, refined or modified until a final category system is reached. Dense descriptions [51] as part of the transcripts will be selected for reconstructive analysis which is based on the inductive principle of grounded theory methodology [41], focusing on *how* participants describe their dealing with depression and *how* they represent themselves. Concepts of positioning [52] and agency [53] will be used heuristically. The reconstructive approach will ensure a deeper understanding of the subjective perceptions of the transformation processes. The interview transcripts will be coded independently by different research assistants. Afterwards the codes will be compared and any discrepancies will be discussed, in order to intersubjectively adjust the coding procedure. If necessary, codes will be clarified until a consensus is reached. Further quality criteria will be ensured by consensual validation of the coding within the research team and within research workshops including researchers from diverse mental health research fields. For transparency, the steps of the analysis process and interims results will be documented in a research diary.

### Data monitoring

Data monitoring is performed by study workers. For each participant, all surveys carried out will be documented in an encrypted file. Archived declarations of consent forms, dropouts and serious adverse events will be documented. All documents will be regularly checked for completeness.

### Ethics

#### Informed consents

Each potential participant will be informed about the goals, the procedures and the potential risks of the study by means of a written document and in plain language. Depending on the recruitment setting, information is provided in person or by phone by trained study workers. Potential study participants receive a copy of the information sheet and will have the opportunity to ask questions before signing the consent form. In case of information by phone, the written documents are send by e-mail in advance. Respondents will be assured that they can refuse to participate or withdraw consent at any time without any adverse consequences. After ensuring that the study information has been properly understood by the participants, they will be asked to sign the consent form.

#### Confidentiality

Signed consent forms will be kept in a locked file cabinet according to Guidelines from Good Clinical Practise E6 (R2) [54]. In addition, an electronic and password-protected file will be created. In this file, pseudonyms (study ID) of the participants and the personal data are stored. The data for analyses will be collected only via the study ID. Only members of the research team have access to the study data and electronic files. Data of participants who withdraw consent will be securely deleted. The study results will be published anonymously, without the possibility of drawing conclusions about individual participants. All data will be archived on the servers of the Department for Psychiatry and Psychotherapy II of Ulm, Germany.

#### Harms

This is an observational study without an intervention and thus, no adverse events are to expect. In the unlikely case of an adverse event occurring, it will documented in an encrypted file and reported during the next research meeting. The evaluation team will be trained and provided with structured instructions to recognize and adequately deal with suspicions or statements of suicide expressed by the study participants.

### Auditing

There are no plans for an independent auditing. The status and progress of the study will be discussed in weekly meetings of the research team. The development of the interview guides and the analysis of the qualitative results will be regularly discussed in qualitative research workshops.

### Protocol amendments

Important changes to the protocol, such as expanding the inclusion criteria or additional recruitment locations, will only be made with the approval of the responsible ethics committee. Changes are immediately updated in the German Clinical Trial Register (DRKS).

## Discussion

While there is broad consensus on the detrimental effects of traditional masculinity orientations on men’s mental health help-seeking behaviour, less is known about male patients’ masculinity orientations in the context of mental health treatment and if and how (possible) changes in masculinity orientations effect treatment and recovery processes and vice versa.

This study aims to address several research gaps: First, investigating the interrelation between masculinity orientations and treatment or recovery processes sheds light on depressed patients’ pathways beyond the help-seeking phase and thus, contributes to a fuller picture of the specific needs of depressed men under mental health treatment [30]. Second, the longitudinal design allows the investigation of changes of masculinity orientations over time and the interplay between masculinity transformations and work-related attitudes on the one hand and mental health, stigmatizing attitudes and service use on the other. Third, the study explores the role of the male patients’ partners and the co-construction of masculinity orientations in the context of depression. Finally, the mixed-methods approach leads to a more comprehensive understanding of the transformation processes by combining robust statistical data on latent classes with a deep insight into the subjective perspectives and contextual factors.

A comprehensive understanding of the transformation processes of masculinity orientations over time including the role of partners in co-constructing these processes can lead to the development of gender-sensitive depression treatment tailored to the unique needs of men with depression. By investigating the partners’ perspectives, the study findings can also identify opportunities for incorporating partners into the therapy process of men with depression. By shedding light on the complex interplay between masculinity orientations, depression, and treatment, this study can promote more effective and successful treatment outcomes and further contribute to reducing the stigma surrounding mental health issues among men and encourage them for mental health service use.

## Data Availability

The quantitative datasets generated during the current study are available from the corresponding author on reasonable request. Full transcripts of the qualitative data generated during the study will not be publicly available, as they might identify individuals, even though they are anonymized.

## Abbreviations

AVEM: Work-related behaviour and experience patterns
CSSRI: Client Sociodemographic and Service Receipt Interview
DMC: Data Monitoring Committee
DSGVO: General Data Protection Regulation
DSS: Depression Stigma Scale
GCP: Good Clinical Practice
MenDe: Masculinity norms and occupational role orientations in men treated for depression
MRNS: Male Role Norm Scale
PHQ-SADS: Patient Health Questionnaire-SADS
TRANSMODE: Transformation of male sex-role orientations and work related attitudes in men with depression

## References

[1] Kilian R, Müller-Stierlin A, Söhner F, Beschoner P, Gündel H, Staiger T (2020). Masculinity norms and occupational role orientations in men treated for depression. PLoS ONE.

[2] Staiger T, Stiawa M, Mueller-Stierlin AS, Kilian R, Beschoner P, Gündel H (2020). Masculinity and help-seeking among men with depression: a qualitative study. Front Psychiatry.

[3] Krumm S, Checchia C, Koesters M, Kilian R, Becker T (2017). Men’s views on depression: a systematic review and metasynthesis of qualitative research. Psychopathology.

[4] McKenzie SK, Collings S, Jenkin G, River J, Masculinity (2018). Social connectedness, and mental health: men’s diverse patterns of practice. Am J Mens Health.

[5] Seidler ZE, Dawes AJ, Rice SM, Oliffe JL, Dhillon HM (2016). The role of masculinity in men’s help-seeking for depression: a systematic review. Clin Psychol Rev.

[6] Zierau F, Bille A, Rutz W, Bech P (2002). The Gotland Male Depression Scale: a validity study in patients with alcohol use disorder. Nord J Psychiatry.

[7] Rutz W, von Knorring L, Pihlgren H, Rihmer Z, Wålinder J (1995). Prevention of male suicides: lessons from Gotland study. Lancet.

[8] Seedat S, Scott KM, Angermeyer MC, Berglund P, Bromet EJ, Brugha TS (2009). Cross-national associations between gender and mental disorders in the World Health Organization World Mental Health surveys. Arch Gen Psychiatry.

[9] Bachmann S (2018). Epidemiology of suicide and the psychiatric perspective. Int J Environ Res Public Health.

[10] Möller-Leimkühler AM, Bottlender R, Strauss A, Rutz W (2004). Is there evidence for a male depressive syndrome in inpatients with major depression?. J Affect Disord.

[11] Hausmann A, Rutz W, Benke U (2008). Frauen suchen Hilfe - Männer sterben! Ist die Depression wirklich weiblich? [Women seek for help - men die! Is depression really a female disease?]. Neuropsychiatr.

[12] Rice SM, Fallon BJ, Aucote HM, Möller-Leimkühler AM (2013). Development and preliminary validation of the male depression risk scale: furthering the assessment of depression in men. J Affect Disord.

[13] Möller-Leimkühler AM (2002). Barriers to help-seeking by men: a review of sociocultural and clinical literature with particular reference to depression. J Affect Disord.

[14] Roche AM, Pidd K, Fischer JA, Lee N, Scarfe A, Kostadinov V (2016). Men, work, and mental Health: a systematic review of depression in male-dominated industries and occupations. Saf Health Work.

[15] Wege N, Li J, Siegrist J (2018). Are there gender differences in associations of effort-reward imbalance at work with self-reported doctor-diagnosed depression? Prospective evidence from the german socio-economic panel. Int Arch Occup Environ Health.

[16] Netterstrøm B, Conrad N, Bech P, Fink P, Olsen O, Rugulies R, Stansfeld S (2008). The relation between work-related psychosocial factors and the development of depression. Epidemiol Rev.

[17] Evans-Lacko S, Koeser L, Knapp M, Longhitano C, Zohar J, Kuhn K (2016). Evaluating the economic impact of screening and treatment for depression in the workplace. Eur Neuropsychopharmacol.

[18] Aronsson G, Theorell T, Grape T, Hammarström A, Hogstedt C, Marteinsdottir I (2017). A systematic review including meta-analysis of work environment and burnout symptoms. BMC Public Health.

[19] Theorell T, Hammarström A, Aronsson G, Träskman Bendz L, Grape T, Hogstedt C (2015). A systematic review including meta-analysis of work environment and depressive symptoms. BMC Public Health.

[20] Theorell T, Hammarström A, Gustafsson PE, Magnusson Hanson L, Janlert U, Westerlund H (2014). Job strain and depressive symptoms in men and women: a prospective study of the working population in Sweden. J Epidemiol Community Health.

[21] Wang JL, Lesage A, Schmitz N, Drapeau A (2008). The relationship between work stress and mental disorders in men and women: findings from a population-based study. J Epidemiol Community Health.

[22] Pyöriä P, Ojala S, Nätti J (2021). Precarious work increases depression-based disability among male employees. Eur J Public Health.

[23] Salk RH, Hyde JS, Abramson LY (2017). Gender differences in depression in representative national samples: Meta-analyses of diagnoses and symptoms. Psychol Bull.

[24] Reeves A, Stuckler D, Suicidality (2016). Economic shocks, and egalitarian gender norms. Eur Sociol Rev.

[25] Rochlen AB, Paterniti DA, Epstein RM, Duberstein P, Willeford L, Kravitz RL (2010). Barriers in diagnosing and treating men with depression: a focus group report. Am J Mens Health.

[26] Gibson K, Cartwright C, Read J (2018). Conflict in men’s experiences with antidepressants. Am J Mens Health.

[27] Seidler ZE, Wilson MJ, Kealy D, Oliffe JL, Ogrodniczuk JS, Rice SM (2021). Men’s dropout from mental health services: results from a survey of Australian men across the life span. Am J Mens Health.

[28] Eggenberger L, Fordschmid C, Ludwig C, Weber S, Grub J, Komlenac N, Walther A (2021). Men’s psychotherapy use, male role norms, and male-typical depression symptoms: examining 716 men and women experiencing psychological distress. Behav Sci.

[29] Sileo KM, Kershaw TS (2020). Dimensions of masculine norms, depression, and mental health service utilization: results from a prospective cohort study among emerging adult men in the United States. Am J Mens Health.

[30] Kwon M, Lawn S, Kaine C (2023). Understanding men’ engagement and disengagement when seeking support for mental health. Am J Mens Health.

[31] Stiawa M, Müller-Stierlin A, Staiger T, Kilian R, Becker T, Gündel H (2020). Mental health professionals view about the impact of male gender for the treatment of men with depression - a qualitative study. BMC Psychiatry.

[32] Westwood M, Black T (2012). Introduction to the special issue of the Canadian Journal of Counselling and Psychotherapy. Can J Counselling Psychother.

[33] Coen SE, Oliffe JL, Johnson JL, Kelly MT (2013). Looking for Mr. PG: masculinities and men’s depression in a northern resource-based canadian community. Health Place.

[34] Bottorff JL, Oliffe JL, Kelly MT, Johnson JL, Carey J (2014). Surviving men’s depression: women partners’ perspectives. Health (London).

[35] Weitkamp K, Bodenmann G (2022). Couples coping together: a scoping review of the quantitative and qualitative evidence and conceptual work across three decades. Front Psychol.

[36] Graham H, Lewin E, Olesen V (1985). Providers, negotiators and mediators: women as the hidden carers. Women, health and healing.

[37] Staiger T, Stiawa M, Müller-Stierlin A, Kilian R, Beschoner P, Gündel H et al. Masculinity and help-seeking amongst men with depression: a qualitative study. Psychiatry Res;submitted. 10.3389/fpsyt.2020.599039 PMC7732518 33329149

[38] Staiger T, Stiawa M, Mueller-Stierlin AS, Kilian R, Beschoner P, Gündel H (2020). Depression und Männlichkeit: Krankheitstheorien und Bewältigung – Eine biografisch-narrative Studie. [Men and depression: illness theories and coping - a biographical narrative study]. Psychiatr Prax.

[39] Baldwin E. A Monte Carlo simulation study examining statistical power in latent transition analysis. [Dissertation]. California: Santa Barbara: University of California; 2015.

[40] Schreier M, Odağ Ö, Mey G, Mruck K (2010). Mixed methods. Handbuch qualitative Forschung in der Psychologie [Handbook of qualitative research in psychology].

[41] Glaser BG, Strauss AL (1999). The discovery of grounded theory: strategies for qualitative research.

[42] Schaarschmidt U, Fischer AW. Arbeitsbezogenes Verhaltens- und Erlebensmuster (AVEM)[Work-related behaviour and experience patterns]. Pearson Assessment & Information GmbH; 2003.

[43] Boerema AM, van Zoonen K, Cuijpers P, Holtmaat CJM, Mokkink LB, Griffiths KM, Kleiboer AM (2016). Psychometric properties of the Dutch Depression Stigma Scale (DSS) and associations with personal and perceived stigma in a depressed and community sample. PLoS ONE.

[44] Kroenke K, Spitzer RL, Williams JBW, Löwe B (2010). The Patient Health Questionnaire somatic, anxiety, and depressive Symptom Scales: a systematic review. Gen Hosp Psychiatry.

[45] Troche S, Rammsayer T (2011). Eine Revision des deutschsprachigen Bem Sex-Role Inventory [A revision of the German adaptation of the Bem Sex-Role Inventory]. Klin Diagnostik u Evaluation.

[46] Roick C, Kilian R, Matschinger H, Bernert S, Mory C, Angermeyer MC (2001). Die deutsche Version des Client Sociodemographic and Service Receipt Inventory - ein Instrument zur Erfassung psychiatrischer Versorgungskosten. [German adaptation of the client sociodemographic and service receipt inventory - an instrument for the cost of mental health care]. Psychiatr Prax.

[47] Götzl C, Staiger T, Stiawa M, Beschoner P, Gündel H, Becker T (2022). Vaterschaft und Depression: familiärer Umgang mit einer depressiven Erkrankung aus Sicht von Vätern – eine qualitative Untersuchung. [Fatherhood and depression: dealing with depression in the family from the fathers’ perspective - a qualitative study]. Psychiatr Prax.

[48] Collins LM, Lanza ST (2010). Latent class and latent transition analysis: with applications in the social, behavioral, and health sciences.

[49] Kuckartz U (2014). Qualitative text analysis: a guide to methods, practice & using software.

[50] Kruse J (2015). Qualitative Interviewforschung. Ein integrativer Ansatz [Qualitative interview studies. An integrative aproach].

[51] Geertz C. Thick description: toward an interpretive theory of cultures. In: Geertz C, editor. The interpreation of cultures. Basic Books; 1973. pp. 311–23.

[52] Korobov N. Reconciling theory with method: from conversation analysis and critical discourse analysis to positioning analysis. Forum Qual Soc Res. 2001:2(3). 10.17169/fqs-2.3.906.

[53] Nentwich J (2009). Zwischen Provokation und Anpassung: Handlungsmächtigkeit als diskursive Positionierung [Between provocation and adaptation: agency as discursive positioning]. Forum: Qualitative Social Research.

[54] European Medicines Agency. Guideline for good clinical practice E6(R2); 2016.

